# Fears Related to Blood-Injection-Injury Inhibit Bystanders from Giving First Aid

**DOI:** 10.5811/westjem.35869

**Published:** 2025-07-08

**Authors:** András N. Zsido, Botond Laszlo Kiss, Julia Basler, Bela Birkas

**Affiliations:** * University of Pécs, Institute of Psychology, Pécs, Department of Cognitive and Evolutionary Psychology, Pécs, Hungary; † University of Pécs, Research Centre for Contemporary Challenges, Pécs, Hungary; ‡ University of Pécs, Szentágothai Research Centre, Pécs, Hungary; § University of Pécs, Medical School, Department of Behavioral Sciences, Pécs, Hungary

## Abstract

**Introduction:**

Prehospital emergency care is vital for saving lives, and increasing bystander involvement can improve survival and recovery. One potential barrier to providing first aid is blood-injury injection (BII) phobia, which affects up to 20% of people, with 3–5% experiencing severe fear. Identifying such barriers may help tailor interventions to encourage willingness to provide first aid.

**Methods:**

We developed and validated the Probability of Giving First-aid Scale (PGFAS), a six-item questionnaire, using the polytomous Rasch Model to assess reliability and validity. The PGFAS was then used to examine how anxiety and disgust-sensitivity related to BII phobia impact the likelihood of providing medical assistance.

**Results:**

Fear of injections and blood draws (β = −0.0987), blood (β = −0.0897) and mutilation (β = −0.1205) significantly reduced the likelihood of giving first aid. However, fear of sharp objects, medical examinations, symptoms of illness, disgust sensitivity, and contamination fear did not have a significant effect.

**Conclusion:**

The Probability of Giving First-aid Scale may serve as a screening tool to identify individuals less likely to provide first aid and could be useful in assessing first-aid training effectiveness. Our findings highlight the importance of preparing first-aid responders and incorporating activities that reinforce helper identity into training programs.

## INTRODUCTION

A short and psychometrically sound questionnaire is needed to assess the willingness of lay people to provide first aid. However, to our knowledge no previous study has proposed a measure that 1) is designed to assess the likelihood of giving first aid in a general sample, rather than of, for example, children^1^,^3^ or nursing students^2^ as in previous studies, and 2) has been systematically tested using psychometric procedures. A questionnaire assessing the self-rated likelihood of providing first-aid could assess the appropriateness of first-aid training in a wide variety of settings (eg, school, workplace). It could also be used to screen specific target populations (eg, caregivers, teachers), to assess how people would react in different situations, such as road accidents or natural disasters, and to identify other factors (eg, personality, emotional response) that might be barriers to intervention.

A key factor in increasing the willingness of people to provide first aid is to identify the barriers that may prevent them from doing so. While helping to educate people about first aid and improving their skills alongside practical application are high priority objectives for organizations like the US Red Cross and American Heart Association, they tend to focus on technical knowledge and not on preparing the individual psychologically to give first aid.^4^,^5^ An earlier study^6^ showed that as little as two hours of technical training, together with activities to support helper identity, can reduce fears for up to two months and consequently increase the likelihood of providing first aid. Similarly, a recent study^1^ emphasized the need to provide first aid and teach first-aid skills to a wider range of people (starting from childhood) and to consider personality-related factors. However, there are few studies investigating the role of underlying— often unconscious—emotional factors, such as fear and disgust, which may act as barriers and prevent people from helping, even if they consciously know they should help and know what to do.^7^–^9^

Both fear and disgust can trigger avoidance behavior, which is an involuntary defensive response initiated upon encountering a potentially harmful object.^9^–^11^ The response is mostly triggered by the perception of a feature or characteristic that is strongly associated with the presence of harm.^12^,^13^ On the one hand, this is a core feature of the defensive survival circuit,^17^,^18^ which is responsible for detecting potential threats, initiating defensive behaviors to avoid them, and making physiological adjustments. The fear response is triggered in parallel with the automatic detection system.^19^ On the other hand, the importance of disgust has also been underscored by the disease-avoidance model,^20^ and also as part of the behavioral immune system.^21^,^22^ Indeed, both fear and disgust have been shown to contribute to the development of distressing contamination-related obsessive thoughts (ie, contamination fear).^23^ From an evolutionary perspective, this is an adaptive response in that it helps to avoid infection, disease, and other pathogens. Avoidance strategies often function to prevent contact with potential contaminants and are associated with fear, disgust, and contamination fear.^26^,^27^ Consequently, both fear and disgust can prevent people from giving first aid; therefore, in the present study we sought to assess their prominence in the willingness to help others.

The purpose of our study was twofold. First, we sought to develop a brief yet reliable and valid measure that could predict the probability of intervening in a potential emergency. Second, we investigated the relationship between blood-injection-injury phobia-related fears, disgust sensitivity, and likelihood of rendering first aid in a non-clinical sample. There are conflicting emotions involved in giving first aid. Seeing someone in need of help activates the approach system as people are generally willing to help others, and they are often driven by curiosity as to what has happened to that individual. In contrast, signs of blood, injury, or disease will also trigger the avoidance system to keep out of harm’s way (due to the possibility of becoming infected or of encountering the same threat that resulted in the injury of the individual in need of first aid). We sought to test whether fear or disgust is a stronger predictor of avoidance in these situations.

## METHODS

### Participants

We targeted the lay population, rather than healthcare professionals, as our goal was to determine what may hold back the average person from intervening in an emergency situation. We recruited participants by posting on social media (Facebook, Twitter, and Instagram), mailing lists, and various discussion forums (Reddit, Lemmy). Respondents completed an anonymous and confidential online survey. We used convenience sampling. We did not record the answers of those who failed to complete the survey.

**Population Health Research Capsule**

What do we already know about this issue?

*Immediate bystander first aid reduces injury severity and mortality, but many people feel unprepared or unwilling to provide first aid*.

What was the research question?

*How do psychological factors, particularly fear of blood and injuries, relate to behavior and willingness to give first aid?*

What was the major finding of the study?

*Fear of blood and injury (β=−0.12, CI −0.21,−0.13, P =.01) predicts less willingness to provide first aid*.

How does this improve population health?

*Identifying fear as a barrier to first aid can help tailor interventions to increase bystander assistance and improve emergency outcomes*.

A total of 906 participants 18–68 years of age (mean 24.83, SD 7.87) volunteered to take part in the study. Table 1 shows the detailed descriptive statistics for the sample. For the psychometric analysis of the Probability of Giving First-Aid Scale (PGFAS), our goal was to recruit as large a sample as possible. The a priori power analysis^28^ for the general linear model used here indicated a minimum required sample size of 791 assuming a small effect size (Cohen f^2^ = .02), power (1-β) = .80, and nine predictors. To ensure the robustness of our analyses and to have a sufficiently large sample for descriptive purposes, we aimed to reach as many participants as possible. Data collection was organized in weekly periods, and recruitment was stopped at the end of the week when the minimum required sample size had been reached.

The dataset used in this study was previously analyzed for a different purpose in one of our earlier studies.^7^ That study focused on behavioral harm avoidance in a healthcare setting, and first aid was not included. The research was approved by the Hungarian United Ethical Review Committee for Research in Psychology and was carried out by the Code of Ethics of the World Medical Association (Declaration of Helsinki). Informed consent was obtained from all participants.

### Questionnaires

Sociodemographic questions included age and (biological) sex. We asked respondents about their previous healthcare-related experiences, education, or practice regarding first aid. These questions assessed whether they had learned first aid; had any healthcare-related education (eg, physician, nurse, paramedic); held a degree in a healthcare-related field; have had healthcare-related jobs; and whether they had to care for a relative for at least one month. These questions were answered on a dichotomous scale (yes or no). Subjective socioeconomic status was measured by calculating the sum score of the questions about 1) the financial status of the family in childhood; 2) support received by parents as a child; and 3) an overall evaluation of their childhood. Questions were rated on 5-point Likert type scales from “1 – lack of funds/no support/very negative” to “5 – plenty of funds/very supportive/very positive.”

The *willingness to perform first-aid* was measured by the PGFAS that we developed for the current study with the help of healthcare professionals. Our goal was to develop a concise yet reliable tool that allows participants to assess the likelihood (ranging from 0–100%) of them performing a specific first-aid action. Items were created based on first-aid guidelines and reviewed for face validity by experts in psychology, first-aid education, and survey design. Minor revisions were made based on expert feedback. Of the questionnaire’s six items, all refer to a step of first-aid behavior considered important by educators and professionals (ie, approach the person, address the person, touch or shake the person, call for help, bandage a wound if necessary, start cardiopulmonary resuscitation). See Table 2 for the questionnaire with instructions. Reliability and internal consistency are detailed in the Results section.

We measured *blood-injection-injury phobia-related fears* using the short, 25-item version of the Medical Fear Survey (MFS).^29^,^30^ The MFS measures an individual’s fear of medical procedures and contexts, including fears related to injections and blood draws, sharp objects, blood and injury, mutilation, and interactions with healthcare professionals. The MFS has five subscales measuring different facets of the concept: injections and blood draws; sharp objects; blood; mutilation; and examinations and symptoms. All items were rated on 4-point Likert-type scales with higher scores indicating higher levels of fear. The internal consistency of the scale was satisfactory (McDonald’s omegas ranged between .79 – .88).

Participants’ *disgust sensitivity* was measured by the revised, 25-item version of the Disgust Scale-Revised (DSR) questionnaire.^31^ The DSR measures disgust sensitivity across three dimensions: core; animal reminder; and contamination-based. Core disgust is primarily concerned with food-rejection response focused on the potential oral ingestion of aversive stimuli (eg, rotting food). Animal-reminder disgust refers to any stimulus or behavior that reminds humans of their animal nature and origin (eg, bodily injury, blood). The contamination disgust factor depicts situations or objects that represent the possibility of coming into contact with a disease. There are 13 true/false items and 12 rated on 3-point Likert-type scales. Higher scores indicate higher levels of disgust sensitivity. (The internal consistency of the scale ranged between .6 – .63). While our mega value for the DSR is below the conventional threshold of 0.7, research indicates that shorter scales or those assessing multifaceted constructs may naturally yield lower reliability coefficients, and previous studies have also found the questionnaire to have low reliability values.^32^ We could have opted to inspect composite reliability (rho), similarly to a study by Olatunji and colleagues,^33^ but as this questionnaire was not the primary instrument under investigation, we opted to report the ω values, as was done with the other questionnaires used.

We assessed *contamination obsessions* and washing compulsions with the Contamination Fear subscale (CF) of the Padua Inventory.^34^ The subscale measures an individual’s fear and avoidance of contamination, typically reflecting obsessive-compulsive concerns about cleanliness, germs, and potential contamination. The CFS is a 10-item, one-factor questionnaire. Each item is rated on a 5-point Likert-type scale. Higher scores indicate more contamination fear. In the present sample, the CFS had satisfactory internal consistency (McDonald’s ω = .85).

### Statistical Analysis Method

First, we tested whether the PGFAS has sound psychometric properties. The unidimensionality (meaning that all six items measure the same underlying construct—the likelihood of providing first aid) was evaluated using confirmatory factor analyses with the diagonally weighted least squares estimator. We assessed the model fit based on the following: the comparative fit index (CFI) and Tucker-Lewis index (TLI), which are used to compare the specified model to the baseline model; the root mean square error of approximation (RMSEA), which evaluates model complexity by penalizing overfitting; and the standardized root mean squared residual index (SRMR) value, which measures the average discrepancy between observed and predicted correlations. The cutoffs for good model fit were CFI and TLI values of .95 or greater^35^ and RMSEA and SRMR values of .08 or lower.^36^ Using multiple indices ensures a balanced evaluation, as each index captures different aspects of model fit.

We also calculated the McDonald omega (conventionally accepted from .7) to check the internal consistency of the scale. We used the polytomous Rasch model to examine both how participants differ in their likelihood of giving first aid (that is, how much of the underlying trait each person has) and how difficult each item is (whether an item is easier or harder to endorse). In the Rasch model, item difficulty refers to the average level of the latent trait required for participants to answer an item in a certain way. For example, items with higher difficulty values require participants with a stronger presence of the trait to choose higher response categories. We report mean values to indicate the average level of the latent trait across the participants for each item, giving us insight into how participants generally perceive the item difficulty.

We used the Rasch model analysis to evaluate whether our questionnaire satisfies the following requirements: the goodness-of-fit (Person separation index > .7) and consistency of the items using the Wright map and infit/outfit measures.^37^,^38^ We used a Mann-Whitney U test (as the PFGAS data are ordinal) to compare male vs female scores for previous experience and knowledge of the PGFAS. The correlation between age, socioeconomic status, and PGFAS scores was observed with the Spearman correlation. Finally, we used the general linear model to test whether the willingness of people to give first aid is more determined by fear- or disgust-related variables. The assumption of normality was not violated. The absolute values of skewness and kurtosis were less than 2 for the PGFAS scale. We performed analyses through jamovi v2.3.28.0 for Windows (https://www.jamovi.org).

## RESULTS

### Questionnaire Characteristics

The one-factor model showed a good fit on our data: x^2^(8)=11.99 *P*=.151, CFI=.99, TLI=.99, RMSE=.02 (90% confidence interval [CI] .00–.05), SMRM=.04. That is, each item depends on a unique latent trait, and the scale can be considered unidimensional. The internal consistency of the test, indicated by the McDonald omega = .89 (95% CI .88–.90), was good. The average interitem correlation was .63 (95% CI .59–.66). The mean score was 46.04 with an SD of 12.58 (range: 6–60), and the median was 49 (MAD robust=11.86). The skewness was −.97 (SD .08), and the kurtosis was .24 (SD .16). Quartile scores were 39 (25^th^ percentile), 49 (50^th^ percentile), and 56 (75^th^ percentile).

### Study Population Characteristics

**Figure 1:**
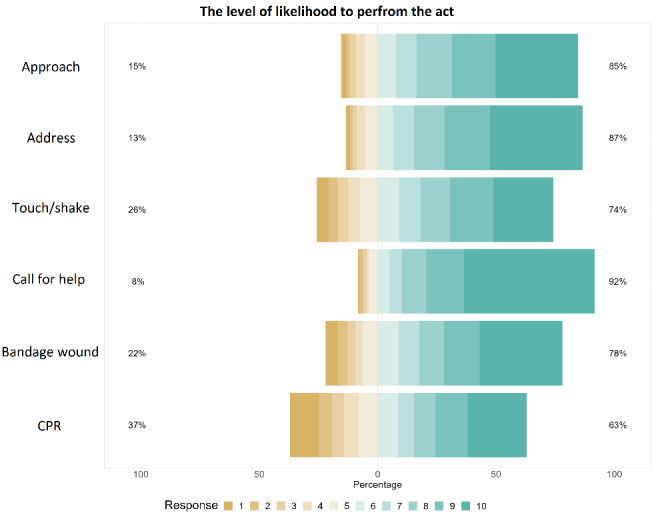
Levels of willingness to render various forms of first aid. *CPR*, cardiopulmonary resuscitation.

Figure 1 presents the weighted proportions of responses for each question regarding levels of willingness to perform first aid. Participants mostly indicated that they would provide first aid. More than half of them (55.3%) would call for help by phone (M = 8.78, 95% CI 8.63 – 8.88); 39.2% reported that they would address the person (M = 8.17, 95% CI 8.03 – 8.32); 35% would bandage a wound (M = 7.50, 95% CI 7.32 – 7.68); 34.8% would approach the person (M = 7.95, 95% CI 7.80 – 8.10); 25.5% would touch/shake the person if unresponsive (M = 7.19, 95% CI 7.01 – 7.37); 25.2% would start CPR (M = 6.47, 95% CI 6.26 – 6.67).

Regarding the demographic variables, we found that males scored slightly higher than females (U=84553, *P*=.03, Cohen d=.096). Further, the correlation between age and PGFAS was positive and weak but significant (rho=.176, *P*<.001, 95% CI .111–.238), indicating that younger people are less likely to give first aid. The correlation between SES and PGFAS was not significant (rho=−.025, *P*=.46, 95% CI −.090–.041). See Supplementary Material 2 for a more detailed analysis of the differences in PGFAS between the grouping variables assessing previous experience.

### Barriers Associated with the Probability of Giving First Aid

**Figure 2:**
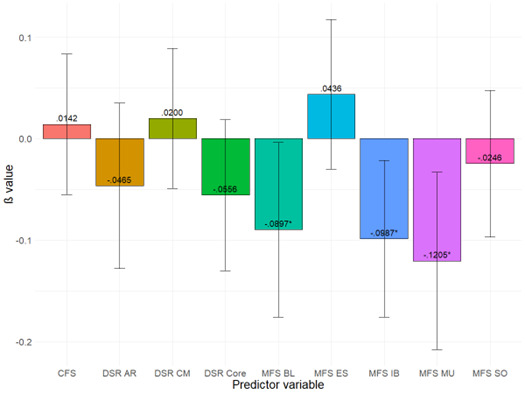
Results of the general linear model analyzing the effects of fear- and disgust-related scales on the Probability of Giving First-Aid Scale total score. Standardized coefficients (β) values are displayed, and error bars represent 95% confidence interval values. β=standardized estimate. Significant results are flagged (* P<.05). *CFS*,contamination fear survey; *DSR*, disgust scale-revised; *AR*, animal reminder; *CM*, contamination fear; *MFS*, medical fear survey; *BL*, blood; *ES*, examinations and symptoms; *IB*, injections and blood draws; *MU*, mutilation; *SO*, sharp objects.

Regarding the predictors of how willing people are to give first aid, the model we tested was significant (F(9, 896)=9.40, *P*<.001, R^2^_a_=.08). This indicated that fear- and disgust-related scores were associated with the PGFAS total score. Figure 2 shows the beta values (see Supplementary Material 3 for more detailed statistical results). Our results show that MFS injection and blood draw, blood, and mutilation scores significantly decreased the probability of giving first aid. In contrast, non-relevant medical fear scales (sharp objects, examination, and symptoms) and disgust-related variables (DS-R and CFS) did not have a significant effect.

## DISCUSSION

We developed a new psychometrically sound questionnaire to measure the PGFAS. The PGFAS identifies individuals who are less likely to engage in first-aid behavior and enables them to overcome the barriers that prevent them from doing so. Further, it might also indicate the appropriateness of this scale as a measure of training effectiveness. Further analysis revealed that the barriers preventing people from providing first-aid included BII-related fears (ie, seeing blood, injections, blood draws, and mutilation). This is consistent with previous studies showing that fear often leads to avoidance behavior.^7^,^8^,^39^ Disgust sensitivity and contamination fear did not emerge as significant predictors in our sample, contrary to what was reported in previous studies.^27^,^40^ Our results support those of previous studies showing the dominance of fear over other emotions^19^ in influencing approach-avoidance behavior. However, it is also possible that disgust only plays a role in individuals with high levels of fear and not in the general (subclinical) population.^41^–^43^

It has been shown that relevant experience or exposure to an object can reduce fear (possibly leading to fear inoculation) and reduce the severity of symptoms and the degree of fear or disgust induced by the next exposure.^8^,^46^–^48^ Our findings show that previous experience and knowledge are key factors in the willingness to provide first aid. Experience is a key factor in both developing^48^,^49^ and overcoming fears.^46^,^51^ Exposure to the object of fear in a safe environment could reduce negative emotions and decrease the likelihood of avoiding the situation or object in the future.^52^ Our results are in line with previous studies emphasizing the importance of teaching first aid starting from an early age^1^ and focusing on personality-related factors in addition to technical knowledge.^6^ These results are important because increasing the likelihood of giving first aid may increase the chances of both survival and full recovery.^1^,^2^ Therefore, bystanders who call for professional help and provide first aid to people in need reduce mortality and morbidity.^45^

## LIMITATIONS

Some limitations of the study are noted here. First, although we used a large sample, the sex imbalance may have confounded the results and could have made groupwise comparison problematic because sex differences are well-documented in specific phobias, including medical fears.^29^,^30^,^53^ Second, the study used convenience sampling through online platforms, which may limit the generalizability of the findings. Although we aimed for a diverse sample, the lack of a representative population—particularly the over-representation of younger adults (18–30 year of age)—may affect the external validity of our results and substantially limits the applicability of our findings to older age groups. Future studies should consider using stratified or random sampling methods to enhance representativeness.

Third, our study relied on self-reported data, which is subject to social desirability bias and individual interpretation of hypothetical emergency situations. Participants’ actual behaviours in real-life emergencies may differ from their self-reported willingness to intervene. Experimental or observational studies could complement self-report measures to provide a more comprehensive assessment. Accordingly, further validation is needed across different populations and settings. Future research should test the scale’s reliability and predictive validity in longitudinal studies and among individuals with varying levels of first-aid training and experience. Finally, other psychological and situational factors, such as personality traits, prior exposure to emergencies, or environmental stressors, may also influence first-aid willingness, and the predictors we used in this study had only a small effect size. Future research should explore a broader range of cognitive, emotional, and contextual variables to provide a more comprehensive understanding of first-aid decision-making.

Despite these limitations, our study contributes to the field by introducing a novel measurement tool and highlighting key psychological barriers to first-aid intervention. Future research should build upon these findings to develop targeted interventions that increase first-aid willingness among the general public.

## CONCLUSION

We developed a brief, self-report measure of the likelihood of providing first aid that can be used as a screening tool to identify those less likely to help someone in need of first aid and to assess the effectiveness of first-aid training. Our findings highlight blood-injection-injury-related fears as a barrier to helping, suggesting that addressing these fears in training could increase willingness to render first aid. Further research is needed to explore additional barriers (such as personal safety concerns unrelated to BII-fears) and develop effective interventions. Despite the limitations and limited prior research on first-aid behavior, these findings offer a promising new approach to studying this area.

## Supplementary Information



## Figures and Tables

**Table 1:** Descriptive statistics of the sample including demographic variables.

Variable	Group		Percentage
Sex	Female		73.1%
	Male		25.7%
	Not responded		1.2%
Studies	Humanities		30.9%
	Natural sciences		10.8%
	Law		3.5%
	Healthcare-studies		22%
	Engineering		14.9%
	Other		17.9%
Care for relative	% Yes		22.4%
Learned first aid	% Yes		83.3%
Healthcare-related studies	% Yes		22%
Healthcare-related qualification	% Yes		6.7%
Healthcare-related job	% Yes		16%

		Median	IQR

Age		22	20–26
SES		12	10–13

*Note*: Participants self-reported their socioeconomic status. Scores could range from 3–15. Percentages for groups labeled with “Yes” indicate the proportion of participants who answered “Yes” to each question.

*IQR*, interquartile range; *SES*, socioeconomic status.

**Table 2:** The Probability of Giving First Aid Scale questionnaire with six items in total. Participants rated how likely they were to perform the given activity on a 10-point Likert-type scale from 0–10% to 91–100%. There were no reverse-keyed items. Score equals the sum of all answers, and higher scores indicate a higher likelihood of giving first aid.

	0–10%	11–20%	21–30%	31–40%	41–50%	51–60%	61–70%	71–80%	81–90%	91–100%
You would approach the person if you were alone.	1	2	3	4	5	6	7	8	9	10
You would address the person.	1	2	3	4	5	6	7	8	9	10
You would touch/shake the person to get more information about their condition.	1	2	3	4	5	6	7	8	9	10
You would call for help by phone.	1	2	3	4	5	6	7	8	9	10
You would bandage a bleeding wound if you had the proper tools.	1	2	3	4	5	6	7	8	9	10
You would start CPR.	1	2	3	4	5	6	7	8	9	10

*Note*. Instruction: Next to each statement, please indicate THE LIKELIHOOD YOU THINK you would do that particular thing if you saw a person lying in the street who you thought might need help.

*CPR*, cardiopulmonary resuscitation.

## References

[1] Huy LD, Tung PT, Nhu LNQ (2022). The willingness to perform first aid among high school students and associated factors in Hue, Vietnam. PLoS One.

[2] Pei L, Liang F, Sun S (2019). Nursing students’ knowledge, willingness, and attitudes toward the first aid behavior as bystanders in traffic accident trauma: a cross-sectional survey. Int J Nurs Sci.

[3] Katona Z, Tarkó K, Berki T (2022). First aid willingness questionnaire for schoolchildren: an exploratory factor analysis and correlation study. Children.

[4] Pellegrino JL, Charlton NP, Carlson JN (2020). 2020 American Heart Association and American Red Cross focused update for first aid. Circulation.

[5] Eisenburger P, Safar P (1999). Life supporting first aid training of the public-review and recommendations. Resuscitation.

[6] Oliver E, Cooper J, McKinney D (2014). Can first aid training encourage individuals’ propensity to act in an emergency situation? A pilot study. Emerg Med J.

[7] Birkás B, Kiss B, Coelho CM (2023). The role of self-reported fear and disgust in the activation of behavioral harm avoidance related to medical settings. Front Psychiatry.

[8] Kiss BL, Birkás B, Zilahi L (2022). The role of fear, disgust, and relevant experience in the assessment of stimuli associated with blood-injury-injection phobia. Heliyon.

[9] Olatunji BO, Smits JAJ, Connolly K (2007). Examination of the decline in fear and disgust during exposure to threat-relevant stimuli in blood-injection-injury phobia. J Anxiety Disord.

[10] Masterson FA, Crawford M (1982). The defense motivation system: a theory of avoidance behavior. Behav Brain Sci.

[11] Castillo-Huitrón NM, Naranjo EJ, Santos-Fita D (2020). The importance of human emotions for wildlife conservation. Front Psychol.

[12] Mermillod M, Droit-Volet S, Devaux D (2010). Are coarse scales sufficient for fast detection of visual threat?. Psychol Sci.

[13] Coelho CM, Araújo AS, Suttiwan P (2022). An ethologically based view into human fear. Neurosci Biobehav Rev.

[14] Van Strien JW, Christiaans G, Franken IHA (2016). Curvilinear shapes and the snake detection hypothesis: An ERP study. Psychophysiology.

[15] Van Strien JW, Franken IHA, Huijding J (2014). Testing the snake-detection hypothesis: larger early posterior negativity in humans to pictures of snakes than to pictures of other reptiles, spiders and slugs. Front Hum Neurosci.

[16] Kuniecki M, Pilarczyk J, Wichary S (2015). The color red attracts attention in an emotional context. An ERP study. Front Hum Neurosci.

[17] LeDoux JE, Daw ND (2018). Surviving threats: neural circuit and computational implications of a new taxonomy of defensive behaviour. Nat Rev Neurosci.

[18] LeDoux JE (2012). Rethinking the emotional brain. Neuron.

[19] LeDoux JE, Pine DS (2016). Using neuroscience to help understand fear and anxiety: a two-system framework. Am J Psychiatry.

[20] Matchett G, Davey GCL (1991). A test of a disease-avoidance model of animal phobias. Behav Res Ther.

[21] Oosterhoff B, Shook NJ, Iyer R (2018). Disease avoidance and personality: a meta-analysis. J Res Pers.

[22] Culpepper PD, Havlícek J, Leongómez JD (2018). Visually activating pathogen disgust: a new instrument for studying the behavioral immune system. Front Psychol.

[23] Woody SR, Teachman BA (2000). Intersection of disgust and fear: normative and pathological views. Clin Psychol Sci Pract.

[24] Oaten M, Stevenson RJ, Case TI (2011). Disease avoidance as a functional basis for stigmatization. Philos Trans R Soc B Biol Sci.

[25] Park JH, Faulkner J, Schaller M (2003). Evolved disease-avoidance processes and contemporary anti-social behavior: Prejudicial attitudes and avoidance of people with physical disabilities. J Nonverbal Behav.

[26] Huijding J, de Jong PJ (2007). Beyond fear and disgust: The role of (automatic) contamination-related associations in spider phobia. J Behav Ther Exp Psychiatry.

[27] Olatunji BO, Etzel EN, Tomarken AJ (2011). The effects of safety behaviors on health anxiety: an experimental investigation. Behav Res Ther.

[28] Faul F, Erdfelder E, Lang A-G (2007). G*Power 3: A flexible statistical power analysis program for the social, behavioral, and biomedical sciences. Behav Res Methods.

[29] Olatunji BO, Ebesutani C, Sawchuk CN (2012). Development and initial validation of the Medical Fear Survey–Short Version. Assessment.

[30] Birkás B, Csathó Á, Teleki S (2022). Confirming the factor structure and improving the screening function of the Medical Fear Survey–short in a Hungarian community sample. Anxiety, Stress Coping.

[31] Olatunji BO, Williams NL, Tolin DF (2007). The Disgust Scale: item analysis, factor structure, and suggestions for refinement. Psychol Assess.

[32] Van Overveld M, de Jong PJ, Peters ML (2011). The Disgust Scale-R: a valid and reliable index to investigate separate disgust domains?. Pers Individ Differ.

[33] Olatunji BO, Adams T, Ciesielski B (2012). The three domains of Disgust Scale: factor structure, psychometric properties, and conceptual limitations. Assessment.

[34] Burns GL, Keortge SG, Formea GM (1996). Revision of the Padua Inventory of obsessive compulsive disorder symptoms: distinctions between worry, obsessions, and compulsions. Behav Res Ther.

[35] Hu L, Bentler PM (1998). Fit indices in covariance structure modeling: sensitivity to underparameterized model misspecification. Psychol Methods.

[36] Browne MW, Cudeck R (1992). Alternative ways of assessing model fit. Sociol Methods Res.

[37] Wang W, Guedj M, Bertrand V (2017). A Rasch analysis of the Charcot-Marie-Tooth neuropathy score (CMTNS) in a cohort of Charcot-Marie-Tooth type 1A patients. PLoS One.

[38] Anselmi P, Vidotto G, Bettinardi O (2015). Measurement of change in health status with Rasch models. Health Qual Life Outcomes.

[39] Armstrong T, Hemminger A, Olatunji BO (2013). Attentional bias in injection phobia: overt components, time course, and relation to behavior. Behav Res Ther.

[40] Cisler JM, Olatunji BO, Lohr JM (2009). Disgust sensitivity and emotion regulation potentiate the effect of disgust propensity on spider fear, blood-injection-injury fear, and contamination fear. J Behav Ther Exp Psychiatry.

[41] Teachman BA, Woody SR (2003). Automatic processing in spider phobia: implicit fear associations over the course of treatment. J Abnorm Psychol.

[42] Tolin DF, Lohr JM, Sawchuk CN (1997). Disgust and disgust sensitivity in blood-injection-injury and spider phobia. Behav Res Ther.

[43] Polák J, Rádlová S, Janovcová M (2020). Scary and nasty beasts: self-reported fear and disgust of common phobic animals. Br J Psychol.

[44] Jánošíková Ľ, Jankovič P, Kvet M (2021). Coverage versus response time objectives in ambulance location. Int J Health Geogr.

[45] Arbon P, Hayes J, Woodman R (2011). First aid and harm minimization for victims of road trauma: a population study. Prehosp Disaster Med.

[46] Coelho CM, Polák J, Suttiwan P (2021). Fear inoculation among snake experts. BMC Psychiatry.

[47] Coelho CM, Purkis H (2009). The origins of specific phobias: Influential theories and current perspectives. Rev Gen Psychol.

[48] Olatunji BO, Ciesielski BG, Wolitzky-Taylor KB (2012). Effects of experienced disgust on habituation during repeated exposure to threat-relevant stimuli in blood-injection-injury phobia. Behav Ther.

[49] Rachman S (1977). The conditioning theory of fearacquisition: a critical examination. Behav Res Ther.

[50] LoBue V, Rakison DH (2013). What we fear most: a developmental advantage for threat-relevant stimuli. Dev Rev.

[51] Lindner P, Rozental A, Jurell A (2020). Experiences of gamified and automated virtual reality exposure therapy for spider phobia: Qualitative study. JMIR Serious Games.

[52] Eaton WW, Bienvenu OJ, Miloyan B (2018). Specific phobias. Lancet Psychiatry.

[53] Kleinknecht RA, Thorndike RM, Walls MM (1996). Factorial dimensions and correlates of blood, injury, injection and related medical fears: cross validation of the Medical Fear Survey. Behav Res Ther.

