# Supplementary material for: Fears Related to Blood-Injection-Injury Inhibit Bystanders from Giving First Aid
Source: West J Emerg Med. 2025 Jul 8;26(4):970–7. doi: 10.5811/westjem.35869 (PMC12342418; doi:10.5811/westjem.35869)

**Supplementary material 1**

We present the results of the general linear model analysing the effects of fear- and disgust-related scales on the Probability of Giving First-Aid Scale (PGFAS) total score. The PGFAS score was used as the dependent variable, while the five subscales of the Medica Fear Survey (MFS), the three subscales of the Disgust Scale-Revised (DSR), and the total score of the Contamination Fear Survey (CFS) was used as independent predictors. The table below displays both point estimates with standard errors (SE) and standard estimates (β) with 95% Confidence Intervals (95%CI). The results of the t-statistics and p-values are also included.

|  | | | | | | | | |
| --- | --- | --- | --- | --- | --- | --- | --- | --- |
|  | | | | | | | **95% CI** | |
| **Predictor** | | **Estimate** | **SE** | **t** | **p** | **β** | **Lower** | **Upper** |
| **MFS** | Injections & Blood draw | -0.3952 | 0.1575 | -2.510 | 0.012 | -0.0987 | -0.1760 | -0.02153 |
|  | Sharp objects | -0.1344 | 0.2014 | -0.667 | 0.505 | -0.0246 | -0.0969 | 0.04772 |
|  | Examinations & symptoms | 0.1568 | 0.1351 | 1.161 | 0.246 | 0.0436 | -0.0301 | 0.11732 |
|  | Blood | -0.3815 | 0.1866 | -2.044 | 0.041 | -0.0897 | -0.1758 | -0.00358 |
|  | Mutilation | -0.3728 | 0.1378 | -2.706 | 0.007 | -0.1205 | -0.2079 | -0.03311 |
| **DSR** | Core | -4.6910 | 3.2014 | -1.465 | 0.143 | -0.0556 | -0.1301 | 0.01888 |
|  | Animal remainder | -2.6434 | 2.3597 | -1.120 | 0.263 | -0.0465 | -0.1278 | 0.03493 |
|  | Contamination | 1.2632 | 2.2237 | 0.568 | 0.570 | 0.0200 | -0.0491 | 0.08911 |
| **CFS** |  | 0.0400 | 0.0990 | 0.404 | 0.687 | 0.0142 | -0.0549 | 0.08329 |
|  | | | | | | | | |

**Supplementary material 2**

We found significant differences in the PGFAS across all five grouping variables assessing previous experience. The figure below shows the central tendencies split for all comparisons. As expected, people who had any previous healthcare-related experience, learned or practiced first-aid previously scored higher than those without such experience or knowledge. (Learned first aid: t(904)=7.61,p<.001, Cohen’s d=.68; Healthcare-related studies: t(904)=7.24, p<.001, Cohen’s d=.58; Healthcare-related degree: t(904)=6.32, p<.001, Cohen’s d=.84; Healthcare-related job: t(904)=6.36, p<.001, Cohen’s d=.58; Care for a relative: t(904)=7.06, p<.001, Cohen’s d=.56).


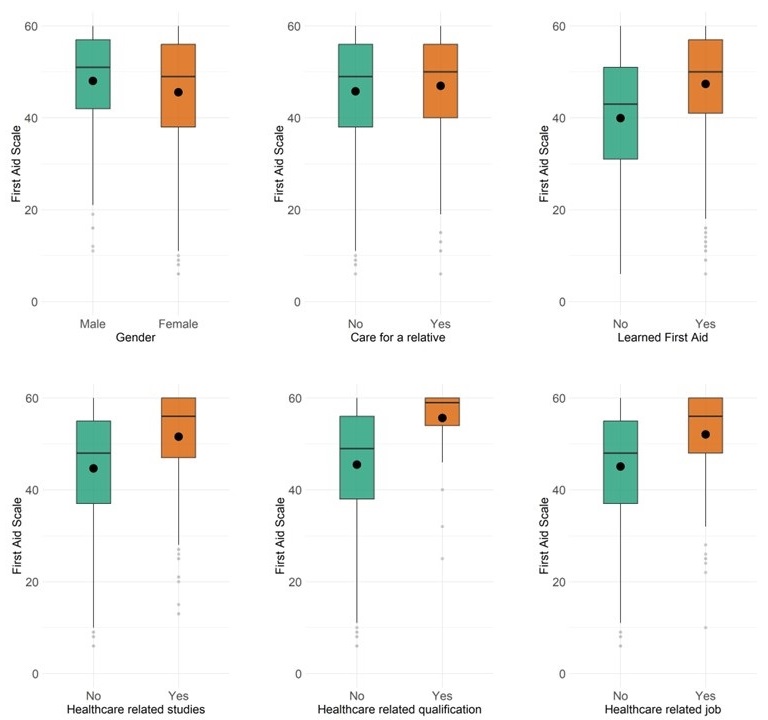

Supplement: Supplementary file 1 [file wjem-26-970-s001.docx]
